# Trigger Factors of Primary Cerebral Hemorrhage Onset: A Case‐Crossover Study

**DOI:** 10.1002/hsr2.71261

**Published:** 2025-09-29

**Authors:** Yun‐Tao Pu, Yan‐Yue Wang, Ya‐Yun Xiang, Miao Zhao

**Affiliations:** ^1^ Department of Neurology The Affiliated Rehabilitation Hospital of Chongqing Medical University Chongqing China; ^2^ Department of Neurology University‐Town Hospital of Chongqing Medical University Chongqing China

**Keywords:** cerebral hemorrhage, risk, strenuous exercise, trigger factor, Valsalva maneuvers

## Abstract

**Background and Aims:**

Compared to traditional chronic risk factors, our understanding of trigger factors for cerebral hemorrhage (ICH) remains relatively primitive. This study aims to identify common trigger factors associated with ICH onset and investigate their diurnal variations in exposure patterns.

**Methods:**

The study population comprised patients with first‐ever primary ICH in Chongqing, Southwest China from January 1, 2024 to December 31, 2024. This is a case‐crossover study, and we compared each patient's exposure during case periods with their own control periods. Through comparative analysis between trigger periods and stable periods, we identified potential trigger factors and further examined diurnal exposure differences between daytime and nighttime.

**Results:**

The study included 1088 ICH patients (mean age 59.6 ± 16.1 years; 632 males [58.1%]). Significant triggers included Valsalva maneuvers (OR 3.05, 95% CI: 2.43–3.83, *p* < 0.001), strenuous exercise (OR 4.38, 95% CI: 3.53–5.45, *p* < 0.001), sudden change of position (OR 3.34, 95% CI: 2.65–4.21, *p* < 0.001), temperature change (OR 1.98, 95% CI: 1.58–2.47, *p* < 0.001), and anger intensity (level 3: OR 5.07, 95% CI: 3.65–7.05, *p* < 0.001; level 4: OR 7.08, 95% CI: 5.08–9.87, *p* < 0.001). Notably, daytime exposures to both Valsalva maneuvers (OR 1.38, 95% CI: 1.04–1.82, *p* = 0.024) and strenuous exercise (OR 3.75, 95% CI: 2.72–5.16, *p* < 0.001) were associated with significantly higher risks compared to nighttime exposures. Conversely, intense anger (level 4) during daytime showed a reduced risk relative to nighttime (OR 0.58, 95% CI: 0.39–0.86, *p* = 0.008).

**Conclusion:**

We identified several triggering factors associated with ICH onset, some of which exhibited distinct diurnal variations in exposure patterns. These findings provide novel insights into the pathophysiology of cerebrovascular rupture and offer evidence‐based references for targeted ICH prevention strategies.

## 1 Introduction

Chronic risk factors for primary cerebral hemorrhage (ICH) have been extensively investigated worldwide and are now well characterized. With a deepening understanding of these risk factors, risk stratification approaches for predicting ICH occurrence have become increasingly feasible. Web‐based risk prediction models incorporating established factors like cardiac disease, hypertension, and diabetes can estimate an individual's ICH risk over several years [1], enabling targeted risk factor modification or therapeutic interventions. Nevertheless, why would a patient with decades‐long hypertension and diabetes develop ICH today? Is short‐term ICH risk predictable or merely stochastic?

Current research reveals distinct diurnal, weekly, and seasonal rhythmic patterns in ICH onset. Substantial evidence demonstrates bimodal daily peaks occurring shortly after morning awakening and/or in the late afternoon [2, 3, 4], with weekly peaks predominantly observed on weekdays, particularly Mondays [5, 6, 7, 8, 9]. Our research team has previously validated these circadian and weekly variations in ICH occurrence, finding strong correlations with population‐specific characteristics related to occupational patterns and lifestyle habits [10, 11]. While traditional risk factors explain population‐level susceptibility through pathophysiological mechanisms, the confirmed temporal patterns of ICH onset strongly suggest nonrandom occurrence. However, predicting the exact timing of ICH events remains exceptionally challenging, even in high‐risk populations, with current preventive paradigms.

Emerging hypotheses propose that vascular events may be precipitated by acute exposures termed trigger factors that directly initiate clinical manifestations [12, 13]. Investigations into trigger effects have established causal links between strenuous exercise, anger, emotional stress, psychological strain, sexual activity, or acute infections with myocardial infarction occurrence [14]. These factors are postulated to qualitatively alter the stability of coronary atherosclerosis through phase transitions, triggering cascades that culminate in plaque rupture and thrombosis. However, the role of trigger factors in ICH pathogenesis remains inadequately substantiated and warrants further exploration.

This study aims to employ a case‐crossover design to elucidate common trigger factors for acute ICH and their diurnal variation in exposure patterns. The findings will advance understanding of short‐term risk determinants for cerebral hemorrhage and inform targeted preventive strategies.

## 2 Methods

### 2.1 Study Design and Participants

In this case‐crossover study, the data were obtained from the medical information systems of two medical institutions: University‐Town Hospital of Chongqing Medical University, and The Affiliated Rehabilitation Hospital of Chongqing Medical University. The study was approved by the Ethics Committee of University‐Town Hospital of Chongqing Medical University, the project's lead organization, and informed consent was obtained.

**FIGURE 1:**
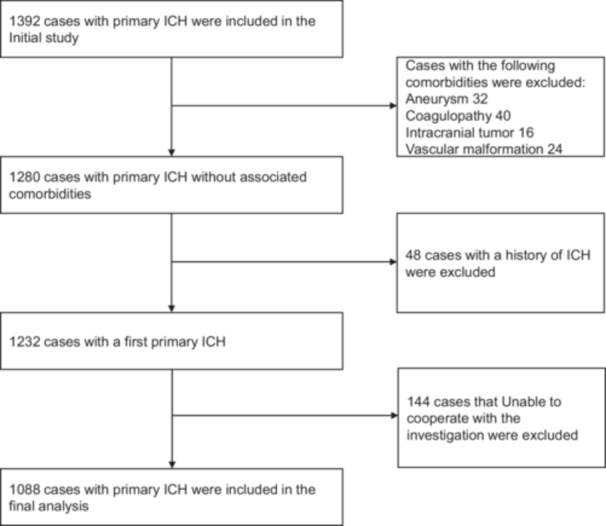
Flow of participants through the screening process.

A total of 1392 patients with primary ICH were initially included in the study, with data collected from January 1, 2024, to December 31, 2024. The study population consisted of patients with the first primary ICH and an age of 18 years or above. A complete cranial computed tomography and/or magnetic resonance imaging was performed on all patients within 24 h of symptom onset. The diagnosis of primary ICH was confirmed by a local neurologist and/or neurosurgeon in accordance with the criteria set forth in the relevant literature [13]. Furthermore, patients must be conscious at the onset of initial neurological symptoms and demonstrate adequate mental status and cognitive function during interviews to reliably recall pre‐stroke events. Cases with comorbidities involving aneurysm, coagulopathy, intracranial tumor, vascular malformation, and a previous history of ICH were excluded from the study. Additional exclusion criteria included cases with indeterminate time of onset and those unable to complete interviews/questionnaires due to other reasons (Figure 1).

### 2.2 Clinical Data

Data were collected through face‐to‐face interviews using a specially designed questionnaire focusing on patients' mental and physical activities, along with environmental temperature fluctuations (including meteorological data from local authorities and patient‐reported indoor‐outdoor temperature differences). The 24‐h period was divided into daytime (9:00–20:00) and nighttime (21:00–8:00). We defined the 0–2 h preceding stroke onset as the trigger phase (primary exposure window for trigger factors), and the preceding 2–24 h as the stable phase. Patients were required to recall and report any notable events during these two phases before ICH onset, specifying exact timing and circumstances. Potential trigger factors included anger, Valsalva maneuvers, sudden change of position, strenuous exercise, alcohol drinking, satiation, and temperature variations.

Potential triggers were identified through comparative analysis of exposure frequencies between trigger and stable phases. Satiation was graded as: Level 1 (mild fullness with preserved appetite), Level 2 (moderate fullness without appetite), Level 3 (severe fullness with nausea/vomiting). Anger intensity was categorized as: Level 1 (slight anger), Level 2 (moderate anger), Level 3 (substantial anger), Level 4 (extreme anger). Significant temperature change was defined as ≥ 10°C decrease within 24 h.

All clinical data, including the patient's sex, age, date and time of onset (hour‐specific), health‐related behaviors, past medical history, and location of hemorrhaging, were recorded. Alcohol Consumption was defined to be those that self‐reported consuming alcohol more than three times per week. Smoking was defined as current regular use (any amount). Hypertension was defined as a self‐reported history of hypertension or blood pressure of 140/90 mmHg or higher. Diabetes was defined as a self‐reported history of diabetes mellitus or an HbA1c level of 6.5% or higher. Coronary heart disease was defined as a self‐reported history of atherosclerotic plaque accumulation within the coronary arterial walls, leading to luminal stenosis (> 50%) and subsequent myocardial ischemia. Hyperlipemia was defined as a self‐reported history of abnormal elevation of plasma lipids, including total cholesterol (TC ≥ 5.2 mmol/L), low‐density lipoprotein cholesterol (LDL‐C ≥ 3.4 mmol/L), triglycerides (TG ≥ 1.7 mmol/L).

### 2.3 Statistical Analyses

We employed a case‐crossover design to investigate acute triggers of ICH onset, as this methodology optimally controls for chronic confounders by using each patient as their own control. Exposure frequencies during the hazard period were compared with control periods to estimate odds ratios (ORs) with 95% confidence intervals (CIs), where OR > 1 indicates increased risk and 95% CI quantifies precision. Key terms were defined as follows: *α* = 0.05 denotes the type I error threshold, and all statistical tests were specified as two‐sided. Analyses comprised pre‐specified primary analysis using conditional logistic regression to calculate ORs for individual trigger factors, and exploratory analyses involving subgroup stratification by diurnal phase and trigger intensity. Interaction effects were tested via product terms, with Holm‐Bonferroni correction applied for multiple comparisons in exploratory analyses. Statistical significance was set a priori at *α* = 0.05. All analyses were conducted using IBM SPSS Statistics v24.0, adhering to SAMPL reporting guidelines.

## 3 Results

A total of 1088 patients with the first primary ICH, with a mean age of 59.6 (16.1) years, and males of 632 (58.1%) were included in this study. The patients were divided into six groups according to the location of the hemorrhage: Basal ganglia 208 (19.1%), Thalamus 200 (18.4%), Lobar 224 (20.6%), Cerebellar 120 (11%), Brainstem 88 (8.1%), Intraventricular 248 (22.8%) (Table 1).

**TABLE 1:** Baseline characteristics.

	ICH patients (*n* = 1088)
Male	632 (58.1)
Age	59.6 (16.1)
Location	
Basal ganglia	208 (19.1)
Thalamus	200 (18.4)
Lobar	224 (20.6)
Cerebellar	120 (11)
Brainstem	88 (8.1)
Intraventricular	248 (22.8)
Medical history	
Hypertension	512 (47.1)
Diabetes	560 (51.5)
Coronary heart disease	496 (45.6)
Hyperlipemia	480 (44.1)
Smoking	552 (50.7)
Alcohol consumption	544 (50)
Data are *n* (%) or mean (SD).	

**Results:** The study included 1088 ICH patients (mean age 59.6 ± 16.1 years; 632 males [58.1%]). Significant triggers included Valsalva maneuvers (OR 3.05, 95% CI: 2.43–3.83, *p* < 0.001), strenuous exercise (OR 4.38, 95% CI: 3.53–5.45, *p* < 0.001), sudden change of position (OR 3.34, 95% CI: 2.65–4.21, *p* < 0.001), temperature change (OR 1.98, 95% CI: 1.58–2.47, *p* < 0.001), and anger intensity (level 3: OR 5.07, 95% CI: 3.65–7.05, *p* < 0.001; level 4: OR 7.08, 95% CI: 5.08–9.87, *p* < 0.001). Notably, daytime exposures to both Valsalva maneuvers (OR 1.38, 95% CI: 1.04–1.82, *p* = 0.024) and strenuous exercise (OR 3.75, 95% CI: 2.72–5.16, *p* < 0.001) were associated with significantly higher risks compared to nighttime exposures. Conversely, intense anger (level 4) during daytime showed a reduced risk relative to nighttime (OR 0.58, 95% CI: 0.39–0.86, *p* = 0.008).

**FIGURE 2:**
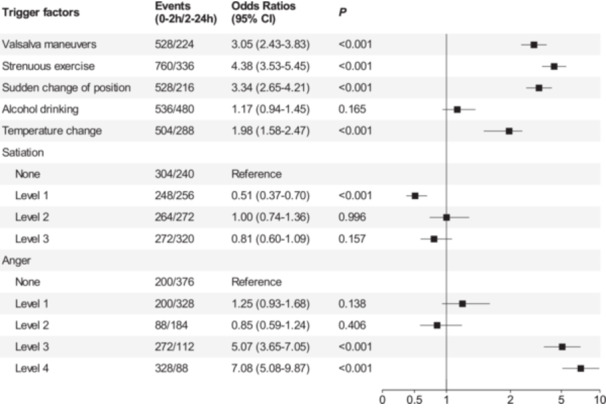
Trigger factors risk ratios during ICH active phase versus stable phase. The squares represent estimated odds ratios (ORs), horizontal lines denote 95% confidence intervals (CIs). *p*‐values indicate comparisons of trigger factors between ICH active phase and stable phase. Control subgroups were defined as: No satiation or anger. *p* < 0.05 was considered statistically significant.

**FIGURE 3:**
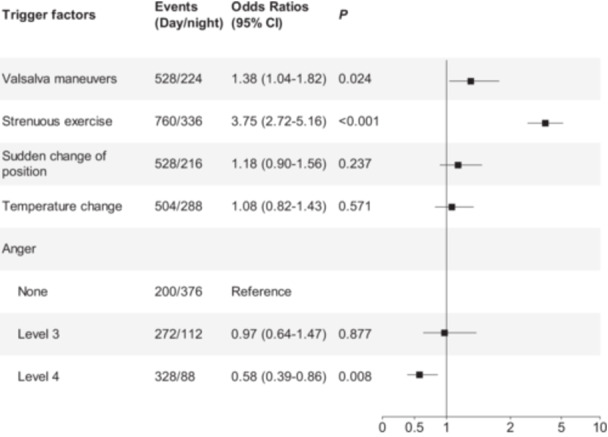
Trigger factors risk ratios for ICH active phase during daytime versus nighttime. The squares indicate estimated odds ratios (ORs), horizontal bars represent 95% confidence intervals (CIs). *p*‐values reflect comparisons of trigger factors between daytime and nighttime ICH active phases. Control subgroups were defined as: No anger. *p* < 0.05 was considered statistically significant.

Compared to the stable period, the risk of ICH occurrence within 2 h after Valsalva maneuvers was 3.05 times higher than in the absence of Valsalva maneuvers (95% CI: 3.53–5.45, *p* < 0.001). Similarly, the risk of ICH increased within 2 h following strenuous exercise (OR 4.38, 95% CI: 3.53–5.45, *p* < 0.001), sudden change of position (OR 3.34, 95% CI: 2.65–4.21, *p* < 0.001), and temperature change (OR 1.98, 95% CI: 1.58–2.47, *p* < 0.001). No increased ICH risk was observed at any intensity level within 2 h after satiation. For anger episodes, grade 1 anger (OR 1.25, 95% CI: 0.93–1.68, *p* = 0.138) and grade 2 anger (OR 0.85, 95% CI: 0.59–1.24, *p* = 0.406) showed no elevated ICH risk within 2 h. However, grade 3 (OR 5.07, 95% CI: 3.65–7.05, *p* < 0.001) and grade 4 anger (OR 7.08, 95% CI: 5.08–9.87, *p* < 0.001) were associated with significantly increased risks (Figure 2). Circadian stratification analysis revealed significantly elevated ICH risks following daytime exposures to both Valsalva maneuvers (OR 1.38, 95% CI: 1.04–1.82, *p* = 0.024) and strenuous exercise (OR 3.75, 95% CI: 2.72–5.16, *p* < 0.001) compared to nighttime occurrences. Conversely, daytime anger intensity (level 4: OR 0.58, 95% CI: 0.39–0.86, *p* = 0.008) showed reduced risk relative to nighttime exposure (Figure 3).

## 4 Discussion

This study found that Valsalva maneuvers, strenuous exercise, temperature change, and sudden change of position within 2 h postexposure were associated with an increased risk of primary intracerebral hemorrhage (ICH), whereas anger required reaching a high intensity to elevate the risk. Daytime Valsalva maneuvers and strenuous exercise increased ICH risk versus nighttime, while daytime intense anger showed lower risk.

Valsalva maneuvers induce transient elevation of intracranial pressure (ICP) by increasing intrathoracic pressure, exerting multifaceted effects on the cerebrovascular system. First, elevated intrathoracic pressure is directly transmitted to the intracranial venous system, causing impaired cerebral venous return, subsequent cerebral blood volume accumulation, and increased ICP [15]. Second, phase II (hypotensive phase) and phase IV (blood pressure rebound phase) of Valsalva maneuvers may trigger dramatic blood pressure fluctuations through sympathetic activation. During phase II, reduced venous return decreases cardiac output and mean arterial pressure, activating the sympathetic nervous system to stabilize blood pressure. In phase IV, simultaneous increases in cardiac output and peripheral vascular resistance drive rapid blood pressure recovery to baseline or higher levels [16]. Such hemodynamic instability is particularly hazardous for hypertensive patients or those with vascular structural abnormalities, potentially precipitating vessel rupture [17]. From a hemodynamic perspective, ICP elevation and blood pressure oscillations induced by Valsalva maneuvers significantly amplify vascular wall shear stress, thereby increasing rupture susceptibility [18].

Strenuous exercise (e.g., anaerobic exercise or high‐intensity interval training) significantly elevates ICH risk within 2 h postexposure, with daytime risk substantially exceeding nighttime levels. Strenuous exercise induces instantaneous systolic blood pressure surges, abruptly increasing mechanical stress on vascular endothelium. Concurrently, exercise‐associated Valsalva maneuvers synergize with blood pressure fluctuations to exacerbate vascular damage [19]. Lactate accumulation from anaerobic exercise may impair mitochondrial function in endothelial cells while promoting pro‐inflammatory cytokine release, thereby compromising vascular repair capacity [20]. The daytime risk predominance correlates with circadian activation of the sympathetic‐adrenal medullary axis. Morning cortisol peaks enhance α‐adrenergic receptor sensitivity, accentuating the morning blood pressure surge [21]. A study confirmed that morning hypertension elevates ICH risk [22].

The increased daytime risk associated with Valsalva maneuvers and strenuous exercise may be related to the circadian rhythm of vascular endothelial activity. Studies have revealed that vascular endothelial activity significantly decreases during daytime, leading to impaired vasodilation capacity and diminished compensatory responses to blood pressure elevation [23]. Furthermore, daytime environmental factors (e.g., noise, social interactions) may synergize with exercise through amplified psychological stress responses [24]. Additionally, the lower frequency of nighttime physical activity could amplify daytime risk disparities, as the vascular system's adaptability to sudden hemodynamic loads remains at a suboptimal level during nocturnal periods.

Sudden change of position (e.g., rapid standing from supine) is associated with elevated ICH risk through mechanisms involving autonomic dysregulation and cerebral perfusion pressure imbalance. Postural changes activate carotid sinus baroreceptors, which normally trigger rapid baroreflex‐mediated adjustments in heart rate and vascular tone to maintain cerebral perfusion. However, in elderly individuals or those with autonomic dysfunction, diminished baroreflex sensitivity delays blood pressure regulation, potentially inducing transient hypertensive responses [25, 26]. Chronic hypertension shifts the cerebral blood flow autoregulation curve rightward, rendering patients more susceptible to cerebral ischemia during abrupt blood pressure drops and vascular rupture during sudden blood pressure spikes [27].

High‐intensity anger (levels 3 and 4) increased ICH risk by 4.95‐fold and 6.4‐fold, respectively, demonstrating a clear dose–response relationship. Anger activates the hypothalamic‐pituitary‐adrenal axis, triggering adrenaline and norepinephrine release within minutes and consequent systolic blood pressure elevation [28]. A study revealed that amygdala hyperactivity during anger directly modulates cardiovascular centers via the locus coeruleus‐noradrenergic pathway [29]. Psychological stress upregulates monocyte chemoattractant protein and intercellular adhesion molecule expression, promoting leukocyte infiltration and vascular wall inflammation [30]. During acute anger episodes, this inflammatory response may be transiently amplified, creating a dual‐hit effect.

This study demonstrates that temperature variation can double the risk of ICH, indicating that abrupt cold exposure may elevate ICH risk, a finding consistent with multiple epidemiological studies. Research suggests that temperature fluctuation exerts greater impact on stroke incidence and mortality than absolute temperature itself. During rapid temperature decline, the human body maintains core body temperature through vasoconstriction to minimize heat dissipation, consequently elevating blood pressure. This abrupt blood pressure surge directly increases vascular wall shear stress, particularly in patients with pre‐existing hypertension or atherosclerosis, predisposing them to vascular rupture [31]. Cold exposure triggers systemic stress responses by activating the sympathetic nervous system, leading to increased secretion of epinephrine and norepinephrine. These hormones induce vasoconstriction and tachycardia, thereby amplifying blood pressure elevation and subsequently enhancing ICH risk [32]. Furthermore, cold stress stimulates endothelial cells to release vasoconstrictive substances like endothelin while suppressing nitric oxide‐mediated vasodilatory effects. This dual mechanism compromises endothelium‐dependent vasodilation and increases vascular fragility [33].

This study has several strengths and limitations. This study utilized a case‐crossover design to effectively control individual confounders through self‐matching. It pioneered systematic analysis of diurnal variations in ICH triggers, informing time‐specific prevention strategies. Incorporation of emotional intensity grading revealed dose–response relationships, enhancing clinical relevance. Recall bias may affect exposure data accuracy, particularly regarding emotional intensity and exercise specifics. Regional sampling limits generalizability to other populations.

## 5 Conclusion

This study found Valsalva maneuvers, strenuous exercise, sudden position changes, temperature shifts, and intense anger as key short‐term triggers for primary ICH. Risks from Valsalva and exercise were higher during daytime, while intense anger risks peaked at night. These findings provide novel insights into ICH pathophysiology, suggesting the need for prevention strategies integrating behavioral interventions and temporal management. Future research should employ real‐time monitoring to validate dynamic trigger effects and develop personalized alert systems for high‐risk populations.

## Author Contributions

**Yan‐Yue Wang:** writing – review and editing, writing – original draft, formal analysis, project administration, data curation, supervision, funding acquisition, methodology, conceptualization, resources. **Ya‐Yun Xiang:** investigation, formal analysis, data curation. **Miao Zhao:** data curation, investigation, formal analysis. **Yun‐Tao Pu:** conceptualization, writing – original draft, writing – review and editing, data curation, software, methodology.

## Ethics Statement

The study was approved by the Ethics Committee of University‐Town Hospital of Chongqing Medical University, approval number: LL‐202212. Informed consent was obtained from all participants, and confidentiality was maintained by not including any personal information or identifiers on the questionnaires.

## Conflicts of Interest

The authors declare no conflicts of interest.

## Transparency Statement

The lead author, Yun‐Tao Pu, affirms that this manuscript is an honest, accurate, and transparent account of the study being reported; that no important aspects of the study have been omitted; and that any discrepancies from the study as planned (and, if relevant, registered) have been explained.

## Data Availability

The data that support the findings of this study are available from the corresponding author upon reasonable request. Yun‐Tao Pu had full access to all of the data in this study and takes complete responsibility for the integrity of the data and the accuracy of the data analysis.
